# Graphical Feature Construction-Based Deep Learning Model for Fatigue Life Prediction of AM Alloys

**DOI:** 10.3390/ma18010011

**Published:** 2024-12-24

**Authors:** Hao Wu, Anbin Wang, Zhiqiang Gan, Lei Gan

**Affiliations:** 1 School of Aerospace Engineering and Applied Mechanics, Tongji University, Shanghai 200092, Chinaganzhiqiang1998@163.com (Z.G.); 2 School of Science, Harbin Institute of Technology, Shenzhen 518055, China

**Keywords:** fatigue life prediction, graphical features, convolutional neural networks, attention mechanism

## Abstract

Fatigue failure poses a serious challenge for ensuring the operational safety of critical components subjected to cyclic/random loading. In this context, various machine learning (ML) models have been increasingly explored, due to their effectiveness in analyzing the relationship between fatigue life and multiple influencing factors. Nevertheless, existing ML models hinge heavily on numeric features as inputs, which encapsulate limited information on the fatigue failure process of interest. To cure the deficiency, a novel ML model based upon convolutional neural networks is developed, where numeric features are transformed into graphical ones by introducing two information enrichment operations, namely, Shapley Additive Explanations and Pearson correlation coefficient analysis. Additionally, the attention mechanism is introduced to prioritize important regions in the image-based inputs. Extensive validations using experimental results of two laser powder bed fusion-fabricated metals demonstrate that the proposed model possesses better predictive accuracy than conventional ML models.

## 1. Introduction

Fatigue is recognized as one of the common causes of in-service mechanical failures in engineering. Therefore, establishing a precise fatigue life model has always been an important concern in fatigue research [1,2]. In this regard, a plethora of models have been formed over the past centuries from the perspectives of solid mechanics, thermodynamics, statistics, and so forth [3,4,5,6,7]. However, it is well known that the fatigue mechanism is elusive and dictated by numerous factors from loading patterns, materials properties, and environmental variables to processing conditions, with certain randomness [8]. As a result, there is still no consensus on developing an accurate, universal, and robust model for fatigue life assessment.

Traditionally, fatigue life models, especially those tailored for metals, are based on researchers’ physical insights and generalization of limited experimental data. As a result, they are nearly semi-empirical solutions, exhibiting limited applicability and accuracy [9]. For instance, in the context of fatigue life prediction for additively manufactured (AM) metals, even though many so-called mechanistic models have formed, the process–microstructure–property–performance relationship is far from being figured out, and reliable predictions that account for the variability of AM processing parameters remain to be attained [10,11]. In his pioneering work, Murakami proposed a two-parameter model that links AM-induced defects and inclusions to fatigue life [12]. By further considering more specific defect characteristics, such as the defect size, morphology, and location, the Z-parameter model and the *X*-parameter models were successively proposed to predict fatigue life [13,14]. Both delve into the link between the geometric parameters of the fatal defects’ interior in materials with fatigue life. These semi-empirical models have advanced the understanding of the fatigue behavior of AM metals. However, due to their empirical nature and the high costs associated with numerical simulations and microstructure characterization, their practical applicability is limited.

Recently, there has been a growing interest in developing machine learning (ML) models for fatigue life prediction [11,15,16,17,18,19,20]. ML models feature a superior efficiency in modeling complex fatigue failure processes under various loading conditions. In this regard, extensive demonstrative instances have been reported. Using random forests (RF) and the kernel extreme learning machine, Gan et al. [16] predicted the fatigue life of diverse metals under uniaxial loading, demonstrating good accuracy, even in the presence of mean stress effects. Lei et al. [11] applied artificial neural networks (ANNs) to estimate the fatigue life of a laser powder bed fusion (LPBF) Hastelloy. Better estimations were observed as compared to those from traditional models. For the low-cycle fatigue issue of 316L stainless steels at elevated temperatures, Jiang et al. [17] developed an ANN-based model that integrates physical constraints into the loss function. The model strikes a good balance between prediction accuracy and computational efficiency. The above works well evidence the promise of ML models in fatigue life analysis.

Essentially speaking, the success of ML models depends on three key factors: training data, ML algorithms, and input features [21]. However, in most application scenarios, the training data are usually provided, and obtaining additional data entails a considerable investment of time and effort. Meanwhile, it is a challenging task for non-specialized researchers to select or develop a suitable machine learning (ML) algorithm based on the characteristics of the target tasks. Given these, optimizing input features is often the primary approach to ensure and improve the performance of ML models.

**Figure 1:**
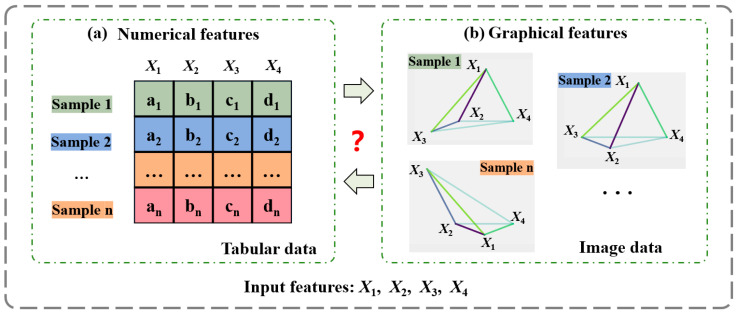
Two feature structures: (**a**) numeric features and (**b**) graphical features.

**Figure 2:**
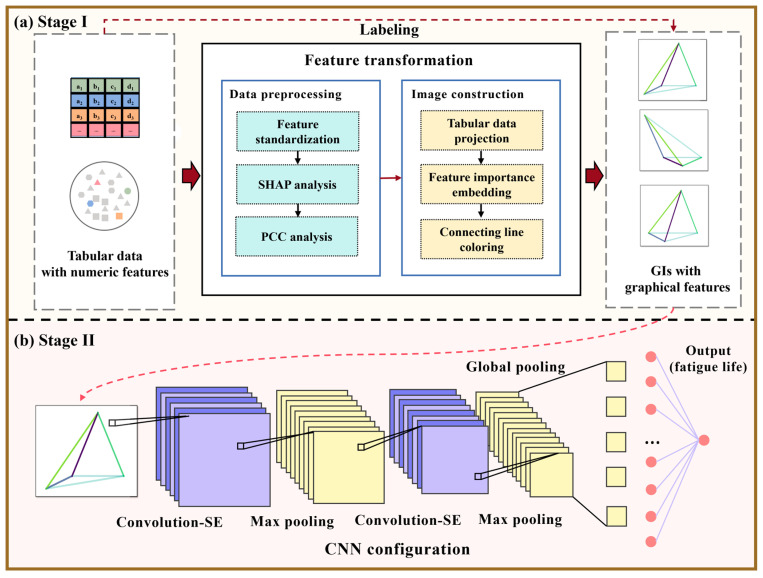
Implementation procedure of the proposed GI-based model: (**a**) the first stage is to preprocess tabular data, that is, convert them into GIs; and (**b**) the second stage is to train an enhanced CNN for predicting fatigue life.

Until now, two kinds of feature structures have appeared: (1) numeric features, typically associated with tabular data, see Figure 1a, and (2) graphical features, represented as clusters of pixel points in images, see Figure 1b. Because of the widespread presence of tabular data and the simplicity of construction, numeric features dominate the current development and application of ML models [22]. Despite this, it is worth noting that graphical features are more informative than numerical features. Specifically, graphical features can provide more visual representations of data patterns, making them easier to capture [23,24]. Also, they are capable of reflecting feature inter-relationships by rationally arranging the coloring, size, and location of related graphical elements [25]. Therefore, it can be anticipated that utilizing graphical features would allow ML models to pursue a better prediction performance, particularly in cases where there are high-dimensional and strongly interactive relationships among features.

Nevertheless, graphical features are difficult to access for most data sources, and there is also a lack of methods enabling the transformation from tabular data into image data. Recently, Elhefnawy et al. [26,27] proposed a sparkling method that can convert tabular data into representative polygons reflecting all feature interrelationships. This method has been well validated by comparing ML models with and without using the converted polygons. However, it is important to note that this method treats all features equally when constructing polygons, ignoring the differences in inter-feature relationships, which could lead to less informative data presentation. Meanwhile, it incurs a high computational budget, owing to its requirement of generating multiple polygons for each data point.

Inspired by the work of Elhefnawy et al. [26,27], this research aims to develop an intuitive and relatively low-budget workflow for transforming tabular data into image data with richer and more visual information. The transformed image data, hereafter called geometrical images (GIs), are anchored in a parallel coordinate system (PCS) and structured according to the calculating results of Shapley Additive Explanations (SHAP) and Pearson correlation coefficient (PCC) analyses. Through the process, GIs are conceptualized as graphs that capture the intrinsic structure and patterns of the data, providing richer and global feature information. Then, the convolutional neural network (CNN), combined with the Squeeze-and-Excitation (SE) attention mechanism [28], is employed as the predictor to learn the feature information inherent in GIs, thereby forming a novel fatigue life model termed the GI-based model. This model not only captures the intensity of fatigue-related factors, but also enables the discernment of hidden structures and patterns within features using geometric graphs, which is challenging to achieve with conventional models using numerical features. Furthermore, the inclusion of an attention mechanism within the GI-based model allows the model to dynamically prioritize important extracted features, thereby enhancing its ability to effectively leverage the information-rich topological geometric graphs. Finally, experimental results of two LPBF-fabricated metals are collected to evaluate this newly presented model.

The remainder of this paper is organized as follows: Section 2 details the GI-based model, with emphases on the GI construction workflow and the basic CNN configuration; Section 3 introduces the experimental databases built for the model evaluation, and then presents the evaluation results with necessary discussions; and Section 4 concludes.

## 2. Proposal of the GI-Based Model

Basically, the richer the information contained in input features, the stronger the predictive capability of ML models. As aforementioned, features for ML models can be represented in numeric and graphical forms. Compared to the former, the latter receives less attention for the time being. However, it indeed offers the benefit of visually representing data structures and provides more accessible information on feature intercorrelations. These features allow for further improving the prediction performance of existing ML models. Recently, Ren et al. [23] used the symmetrized dot pattern images of vibration signals, instead of raw numerical signal sequences, to train a CNN-based model for bearing fault diagnosis. It was experimentally demonstrated that the graphical features embedded in these images can help capture the interconnections between different fault characteristics, leading to an enhanced diagnostic accuracy.

Given the advantages of graphical features, this work proposes converting widely available tabular data into GIs, thereby transforming numeric features into corresponding graphical representations. To this end, the presented GI-based model is equipped with a two-stage implementation process. As illustrated in Figure 2a, with the aid of SHAP and PCC analyses, Stage I manages to transform tabular data with numerical features into GIs with graphical features, during which operations of feature projection, feature importance ranking, and feature intercorrelation quantification are conducted in order. This stage facilitates visualizing the interplays among features and helps characterize the importance of multiple features for fatigue life modeling. Then, as shown in Figure 2b, Stage II involves a conventional training–validating–predicting routine conducted on an enhanced CNN using GIs as inputs. More details are described in the following sections.

### 2.1. Implementation Stage I: Transformation from Tabular Data into GIs

**Figure 3:**
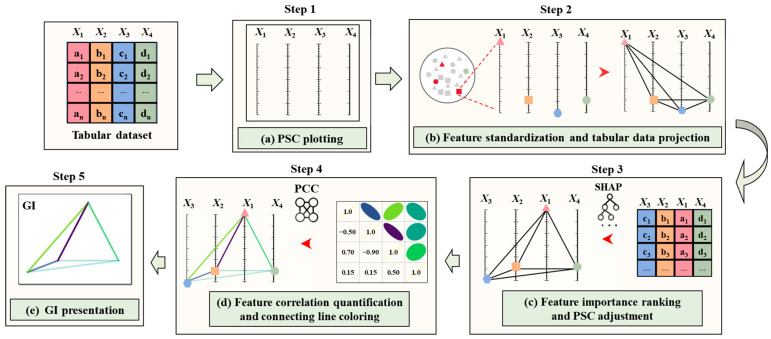
Workflow of constructing GIs based on given tabular data.

A dataset generally comprises a mass of data instances and a series of distinct features, which can store three types of information: (i) the intensity of each feature of every data instance, (ii) the importance of each feature relative to the output variables, and (iii) the underlying interrelationships among features. In tabular data, numerical features can only express the first type of information explicitly. Fatigue, however, is a complex problem characterized by strong interactions between multiple influencing factors. Hence, to fully incorporate all three types of information into the learning of the GI-based model, it is suggested to transform tabular data into GIs by the workflow depicted in Figure 3. This transformation enables the model to capture not only the intensity and importance of features but also the complex interrelationships between them, which traditional numerical data representations cannot fully express.

The specific transformation steps include the following:

Step 1: plot a PCS from the beginning by drawing a set of parallel vertical axes (see Figure 3a);

Step 2: standardize the feature intensities of all given tabular data, and then project each data instance onto the PCS, where feature locations are connected pairwise via black lines (see Figure 3b);

Step 3: rank the importance of features for fatigue life prediction based on the SHAP analysis, and the PCS axes is rearranged accordingly (see Figure 3c);

Step 4: quantify the intercorrelation strength among features based on the PCC analysis and subsequently, color the connecting lines among features according to their intercorrelation strengths (see Figure 3d);

Step 5: remove the PCS, retaining a colored geometry, i.e., the so-called GI (see Figure 3e).

Detailed introductions are provided below.

#### 2.1.1. Feature Standardization and Tabular Data Projection

For transforming tabular data into GIs, a benchmark image format that can compatibly accommodate their basic characteristics is needed. In the developed workflow, the imaged-based PCS, shown in Figure 3, is used on account of its simplicity in construction. Then, the feature intensities for all given tabular data can be standardized. Suppose a tabular dataset consisting of *n* data instances and *m* features (*X*_1_, *X*_2_ … *X*_*m*_), and a PCS where the number of axes equals that of features. Feature standardization can be given as follows [29]:

(1) $$Z_{ij}=\frac{X_{ij}-\bar{X_{j}}}{\delta_{j}},j=1,\ldots ,m,i=1,\ldots ,n$$

where $X_{ij}$ is the *j*th feature value of *i*th data instance, $Z_{ij}$ is the mean standardized value of the *i*th sample for the *j*th feature, $\bar{X_{j}}$ is the mean value of the *j*th feature across all data instances, and $\delta_{j}$ is the standard deviation of the *j*th feature.

With feature standardization, the scale and distribution discrepancies in data can be minimized, facilitating better training of ML models. After that, it is feasible to project the given tabular data onto the PCS. In more detail, as shown in Figure 3b, the feature locations of each data instance can be first plotted on their respective feature axis in the PCS. Then, each feature location can be connected with others via black lines. This practice outputs a single image visually showcasing the feature intensities of considered tabular data. It is worth mentioning that the intercorrelation between any two features can also be graphically described by their connecting line.

#### 2.1.2. Feature Importance Ranking Based on SHAP Analysis

To consider the importance of features for fatigue life prediction, the SHAP analysis, derived from cooperative game theory, is introduced during the course of GI construction. In the fatigue domain, the SHAP value is commonly used to quantify the contribution of each feature to accurately predicting a specific data instance [30]. By averaging the SHAP values across all data points, the overall importance of each feature can be determined. For a single feature, the SHAP value can be computed as follows [30]:

(2) $$\mathrm{SHAP}(X_{i})=\sum_{S\subseteq F\backslash \{X_{i}\}}\frac{|S|!(|F|-|S|-1)!}{|F|!}[f(S\cup X_{i})-f(S)]$$

where *F* is the feature set including all features, *S* is a feature subset excluding the feature *X_i_*, $f(S\cup X_{i})$ is the output function with *S* and *X_i_* as inputs, and f(S) is the output function without concern for *X_i_*. Equation (2) calculates the average contribution of *X_i_* under the consideration of all allowable feature subsets.

By applying the SHAP analysis on all investigated features, their importance for fatigue life prediction can be revealed. To embed this information into GIs, the PCS can be further adjusted by rearranging the order of the feature axes from left to right, according to the descending order of feature importance ranking. This rearrangement places important features closer together in the PCS, which better aligns with the CNN’s focus on local feature extraction, enabling it to more effectively capture local patterns. Other rearrangements are also usable, provided that the information on feature importance ranking is not lost and can be fully extracted by ML models.

#### 2.1.3. Feature Correlation Quantification Based on PCC Analysis

In the previous contents, the information about the feature intensity and feature importance ranking are considered. Here, the interrelationships among features are further taken into account in the GI construction workflow by employing the PCC analysis [31]. The PCC analysis aims to measure the correlation between two features, providing a value from −1 to 1, and each PCC value indicates a certain degree of feature correlation strength, calculated as follows [31]:

(3) $$\mathrm{PCC}(Y,W)=\frac{\sum_{i=1}^{n}\left(Y_{i}-\bar{Y}\right)(W_{i}-\bar{W})}{\sqrt{\sum_{i=1}^{n}{\left(Y_{i}-\bar{Y}\right)}^{2}}\sqrt{\sum_{i=1}^{n}{\left(W_{i}-\bar{W}\right)}^{2}}}$$

where *Y* and *W* represent two different features; and *Y_i_* and *W_i_* represent the *i*th data points of the two features, respectively. $\bar{Y}$ and $\bar{W}$ denote the mean values of *Y* and *W*, respectively, and *n* represents the number of data instances considered.

As described in Equation (3), the PCC value quantitively measures the correlation strength between two features. Meanwhile, as noted in Section 2.1.1, the connecting line between a pair of features graphically represents their correlations. Considering these, the PCC value is leveraged in the work to guide the coloring of the connecting line between any two features, so as to visualize their correlation strength explicitly, see Figure 3d. The coloring function is defined as follows:

(4) $$\mathrm{Line}\;\mathrm{color}(r,g,b)=v(\frac{\mathrm{PCC}(Y,W)+1}{2}\times l)$$

where *v* denotes a color spectrum, *l* denotes the number of color levels, and *r*, *g*, and *b* refer to the values in the three-primary-color channels, respectively. After several trials, the viridis color spectrum was selected for its perceptual uniformity and effective distinction between color levels, addressing the need for the clear and accurate visualization of correlation strengths.

**Figure 4:**
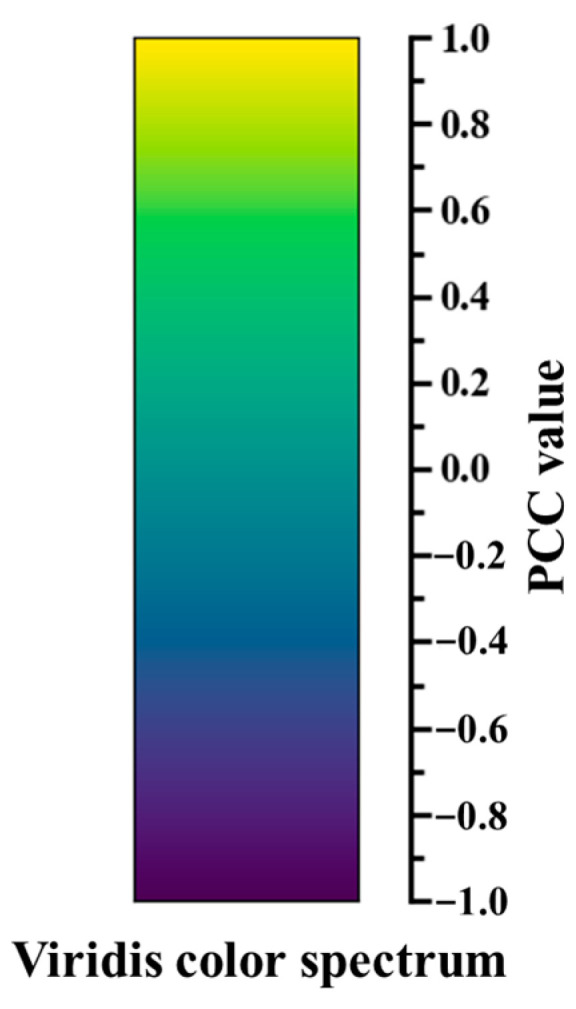
Viridis color spectrum and corresponding variation of PCC value.

As depicted in Figure 4, this color spectrum shows a smooth transition of colors from purple to yellow, in line with the continuous variation of PCC value from −1 to 1. Using Equation (4), the correlation strength between any two features can be visualized via relevant colored lines, with pure purple and yellow representing the extreme PCC values of −1 and 1, respectively. More importantly, the PCC value can be calculated based on all available data, including those unlabeled ones, which maximizes the utilization of available data information. It is worth noting that while PCC was chosen for its simplicity and ease of use, it is not the only metric for representing the correlation between features. In practice, the choice of correlation metric can be made according to the specific requirements of the application.

The last step of the developed GI construction workflow is to remove all non-essential information for life modeling, i.e., the manually generated PCS. This ultimately outputs a colored geometry, called GI, for each instance of tabular datasets.

In summary, with the developed GI construction workflow, tabular data can be represented in a more visual way with informative graphical features, not only reflecting the feature intensity, but also providing supplementary information on the importance of each feature and the correlation strengths among them. All this information is valuable for ML models, especially when addressing complex prediction tasks involving multiple intercorrelated factors. Note here that each constructed GI must be labeled according to the original tabular datasets for the supervised learning of fatigue life.

### 2.2. Implementation Stage II: Fatigue Life Prediction via an Enhanced CNN

The implementation Stage II of the proposed GI-based model involves training an ML predictor with these constructed GIs as inputs. Here, CNN, more specifically the 2D-CNN, is chosen as the predictor, because of its suitability in addressing image-based inputs.

A basic 2D-CNN configuration generally includes four types of neural network layers: (1) the convolutional layer, serving as the core of CNNs to extract important graphical features from input images by multiple filters; (2) the activation layer, which introduces non-linearities into CNNs to enable the learning of complex data patterns; (3) the pooling layer, devised to reduce the dimensionality of input images; and (4) the fully connected layer that focuses on correlating extracted graphical features with targeted variables. For detailed introductions of the 2D-CNN, please refer to Refs. [32,33,34].

In addition to the above neural network layers, the SE attention mechanism is additionally utilized to configure the CNN predictor to enhance its accuracy [28,35]. The attention mechanism can enforce ML predictors to focus on the important parts of input data, helping arrest critical data information. Among diverse attention mechanism techniques, the SE attention mechanism can achieve a good trade-off between enhancing accuracy and maintaining computational efficacy. It works with two operations: the squeeze and excitation operations. In the squeeze operation, the global average pooling is applied to each channel of input images to produce a representative vector. Afterward, the excitation operation scales the representative vector within the range of 0 to 1 and then adopts it to adjust the importance of each channel in the original input images during modeling.

**Figure 5:**
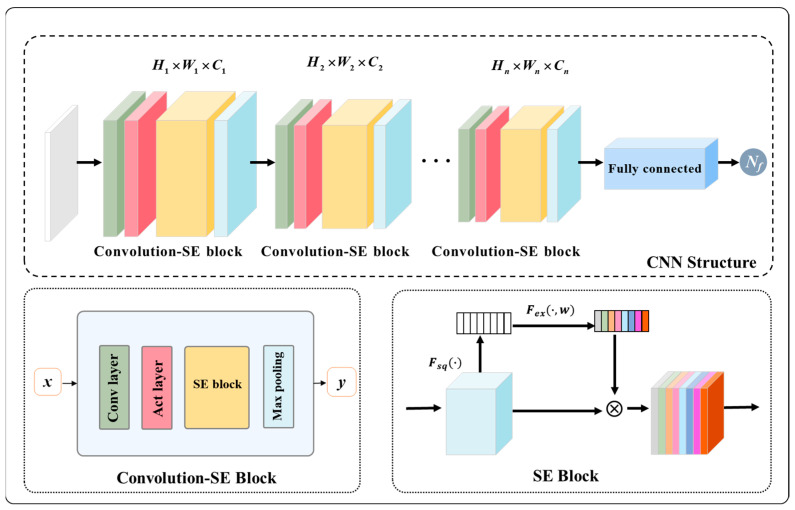
Illustration of a typical 2D-CNN structure enhanced with the SE attention block. The symbol $F_{sq}(\cdot )$ represents the squeeze operation, and the symbol $F_{ex}(\cdot ,w)$ represents the excitation operation within the SE block.

As illustrated in Figure 5, the input dimensions *H*_1_ × *W*_1_ × *C*_1_ (Height, Width, and Channels) are processed through Convolution-SE blocks, each consisting of a convolutional layer, activation layer, SE block, and max pooling layer. The SE block can enhance feature recalibration by emphasizing informative channels. Dimensions are progressively reduced to *H_n_* × *W_n_* × *C_n_* through these blocks. Finally, the output is passed through a fully connected layer to predict fatigue life (*N_f_*).

Through the above process, the GI-based model for additive fatigue prediction is constructed. From a fatigue perspective, the proposed model offers the following advantages: (1) Fatigue-related factors exhibit strong interactions, and the GI-based model captures these complex, multidimensional relationships by transforming conventional numerical features into graphical representations. These graphs not only represent the intensity of the features, but also illustrate high-dimensional intrinsic connections and interactions among the features [26], thereby enriching the feature information used for model training and enhancing its predictive capability. (2) The multi-layer convolutional structure of CNNs effectively extracts both local and global features from the constructed topological geometric graphs [34], potentially identifying patterns that are less apparent in conventional numerical data representations. The GI model provides a complementary perspective to traditional tabular data approaches. (3) Using techniques like feature maps [32], the GI model allows for a more comprehensive understanding of the complex interactions among fatigue-related parameters, offering new insights into the underlying mechanisms of fatigue behavior in materials.

## 3. Model Evaluation

This section evaluates the prediction performance of the proposed GI-based model and compares it with three conventional ML models. In addition, ablation studies are performed to examine the benefits of GIs and the SE attention mechanism in improving prediction accuracy. Quantitative evaluation results are given using the Mean Squared Error (*MSE*) and Coefficient of Determination (*R*^2^) as the accuracy indexes as follows:

(5) $$MSE=\frac{1}{n}\overset{n}{\underset{i=1}{\sum }}{\left(y_{i}-{\hat{y}}_{i}\right)}^{2}$$

(6) $$R^{2}=1-\frac{\sum_{i=1}^{n}{\left(y_{i}-{\hat{y}}_{i}\right)}^{2}}{\sum_{i=1}^{n}{\left(y_{i}-{\bar{y}}_{i}\right)}^{2}}$$

where $y_{i}$ is the *i*th actual value, ${\hat{y}}_{i}$ is the *i*th predicted value, ${\bar{y}}_{i}$ is the average of actual values, and *n* is the number of studied data instances.

### 3.1. Experimental Databases

Based on experimental results from the literature [36,37,38,39,40,41,42,43], two databases were established for the model evaluation. These databases were selected based on the completeness of the manufacturing process information provided and the consistency of the material types. One of the databases includes 103 results generated for LPBF 316L stainless steel, and the other contains 77 results for LPBF AlSi10Mg alloy. Specimens in all experiments were fabricated using gas-atomized metal powders via the LPBF technology. For the LPBF 316L stainless steel, gas-atomized 316L powder with predominantly spherical particles was processed using an EOS M-290 system (Krailling, Germany) equipped with a Yb-fiber laser. For the LPBF AlMg10Si alloy, gas-atomized AlMg10Si powder was used, with specimen fabrication completed using the SLM Solutions SLM 280 (Lübeck, Germany) and EOSINT M-280 systems (Krailling, Germany). All fatigue tests were conducted under uniaxial load-controlled conditions at ambient temperature, with LPBF 316L stainless steel tested with a load ratio of *R* = 0.1 and LPBF AlMg10Si alloy with *R* = −1 and 0.1. More experimental details can be found in Refs. [36,37,38,39,40,41,42,43].

**Figure 6:**
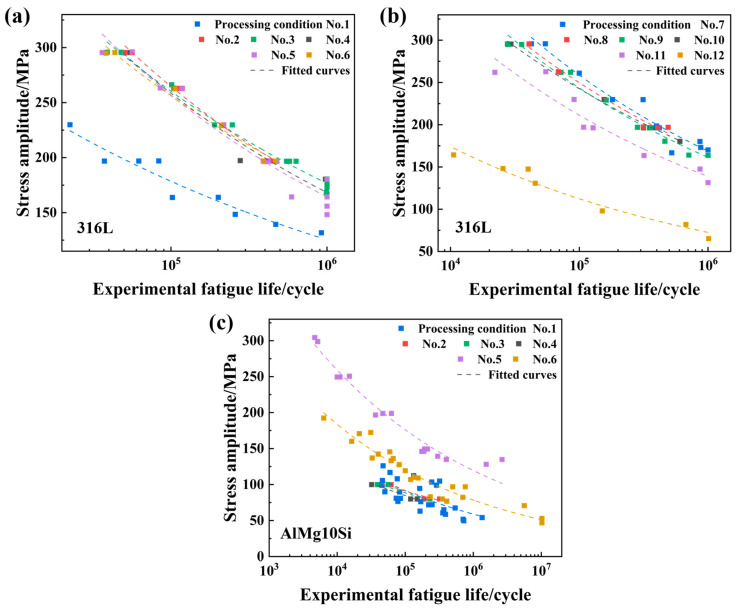
Distribution of collected experimental data in the representation of *σ_a_* vs. *N_f_* : (**a**) processing conditions No. 1–6 for LPBF 316L stainless steel, (**b**) processing conditions No. 7–12 for LPBF 316L stainless steel, and (**c**) processing conditions No. 1–6 for LPBF AlSi10Mg alloy.

Seven variables are concerned in the two established databases, which are the laser power (*P*), scanning speed (*ʋ*), powder layer thickness (*t*), hatch spacing (*h*), applied stress amplitude (*σ_a_*), stress ratio (*R*), and the target variable—fatigue life (*N_f_*). Moreover, Table 1 tabulates various processing conditions existing in both databases. Note here that *h* is fixed for LPBF 316L stainless steel, and no specific value can be found in the source articles. Figure 6 displays the distribution of collected experimental data by plotting *σ_a_* vs. *N_f_* under different processing conditions. It is visible that the fatigue properties of both LPBF 316L stainless steel and LPBF AlMg10Si alloy are highly dependent on processing conditions.

### 3.2. Preparation of GI Databases

**Figure 7:**
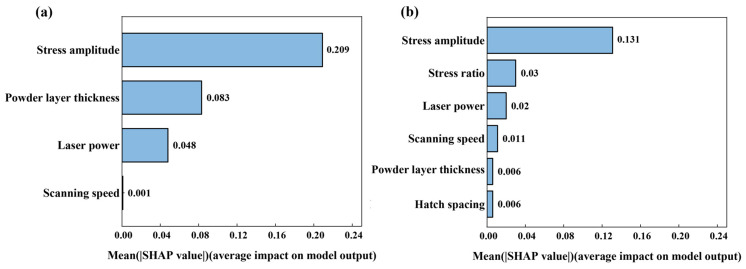
Feature importance ranking by the SHAP analysis for (**a**) LPBF 316L stainless steel, and (**b**) LPBF AlSi10Mg alloy.

**Figure 8:**
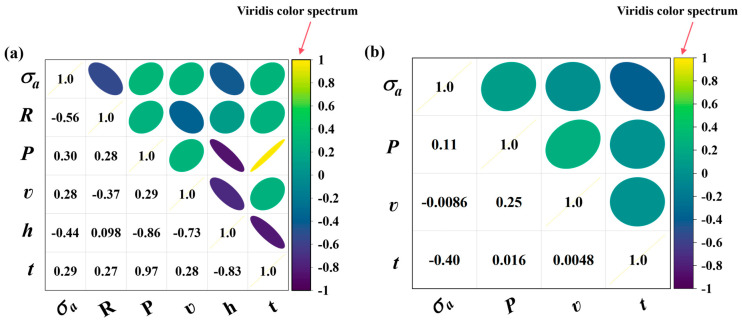
Feature intercorrelation strengths calculated by the PCC analysis for (**a**) LPBF AlSi10Mg alloy, and (**b**) LPBF 316L stainless steel.

**Figure 9:**
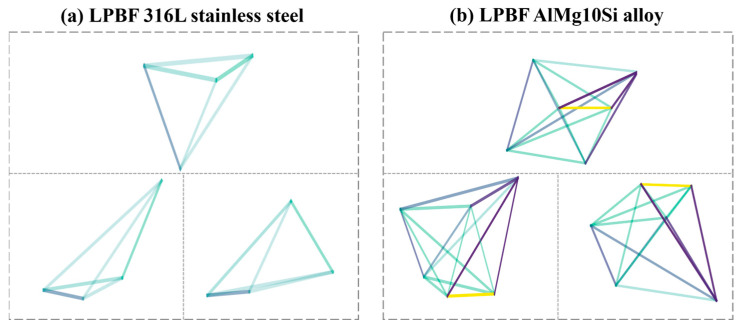
Illustrative GIs for (**a**) LPBF 316L stainless steel, and (**b**) LPBF AlSi10Mg alloy.

To apply the GI-based model, the previously established databases need to be reorganized according to the developed GI construction workflow. Before that, the input features are set as follows: (*P*, *ʋ*, *t*, *σ_a_*) for LPBF 316L stainless steel, and (*P*, *ʋ*, *h*, *t*, *R*, *σ_a_*) for LPBF AlMg10Si alloy. Then, feature standardization can be applied to both databases, followed by the SHAP and PCC analyses, as described previously. Figure 7 presents the SHAP analysis results, where the mean SHAP value quantifies the average contribution of each feature to the model’s output. A higher mean SHAP value indicates greater overall feature influence. Figure 8 displays the PCC analysis results, illustrating the intercorrelation strengths among various features. Based on these results, GIs can be constructed with the workflow outlined in Figure 2, thereby forming two GI databases prepared for LPBF 316L stainless steel and LPBF AlMg10Si alloy, respectively. Several illustrative GIs are given in Figure 9.

### 3.3. Evaluation Results

#### 3.3.1. Comparison with Conventional ML Models

In this subsection, the proposed GI-based model is evaluated in comparison with four conventional ML models, i.e., the classical back-propagation neural network (BPNN) model [44], the 1D-CNN model [45], the Support Vector Regression (SVR) model [46], and the RF model [47]. All the conventional ML models operate on tabular data. In addition, to avoid occasionality, each evaluated model was run 10 times, and the average prediction performance is presented for the model comparison. For all evaluated models, the training, validation, and prediction data were randomly selected in a fixed division ratio of 7:1:2, based on the databases of LPBF 316L stainless steel and LPBF AlSi10Mg alloy.

Table 2 summarizes the optimizing ranges of hyperparameters of each evaluated model, and also lists corresponding optimization outcomes. All hyperparameters involved were optimized using either the grid search method or the trial-and-error method. In addition, for the BPNN model, the Adam optimizer was employed to drive the model training [48], the maximum number of training iterations was set to 5000, and the training process stopped if the validation accuracy did not improve over 300 consecutive iterations. The same training scheme was applied to both the 1D-CNN and GI-based models.

**Figure 10:**
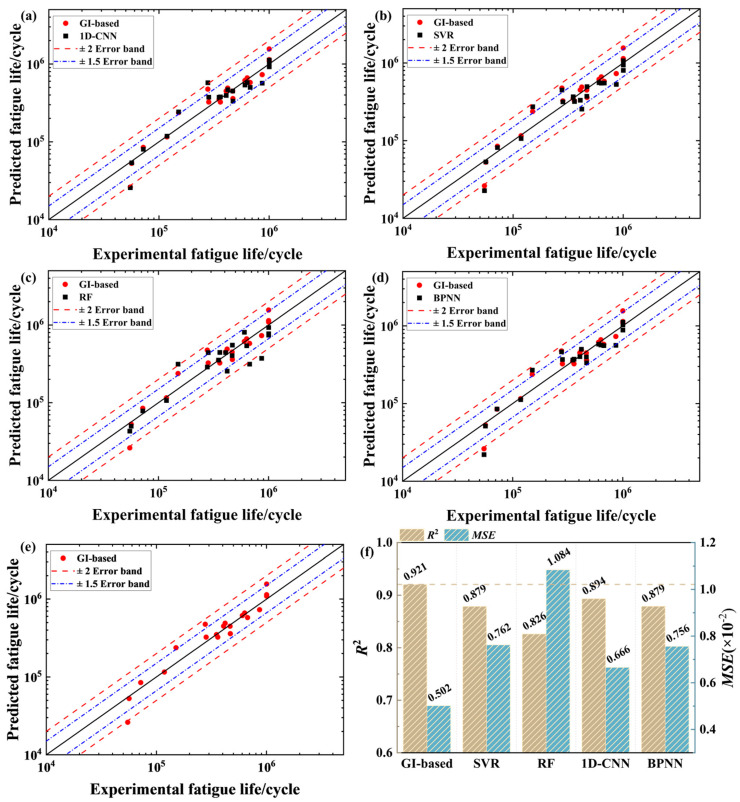
Comparison of experimental and predicted lives for LPBF 316L stainless steel based on (**a**) 1D-CNN, (**b**) SVR, (**c**) RF, (**d**) BPNN, and (**e**) GI-based. (**f**) *R*^2^ and *MSE* values for each model.

Figure 10 displays the comparison of experimental lives and predictions from different models for LPBF 316L stainless steel. The figure shows that most predictions can lie within the ×2 error band, indicating that all evaluated models have an acceptable accuracy. Moreover, it is visible that predictions from the GI-based model are closer to the diagonal line, with the majority falling inside the ×1.5 error band. As shown in Figure 10e, overall, it demonstrates a better accuracy as compared to other evaluated models. This result confirms that the GIs, transformed from tabular data, are useful inputs in promoting the accuracy of fatigue life prediction. They can give real benefits to the GI-based model by representing tabular data more visually and explicitly, providing more informative graphical features correlated to fatigue life.

**Figure 11:**
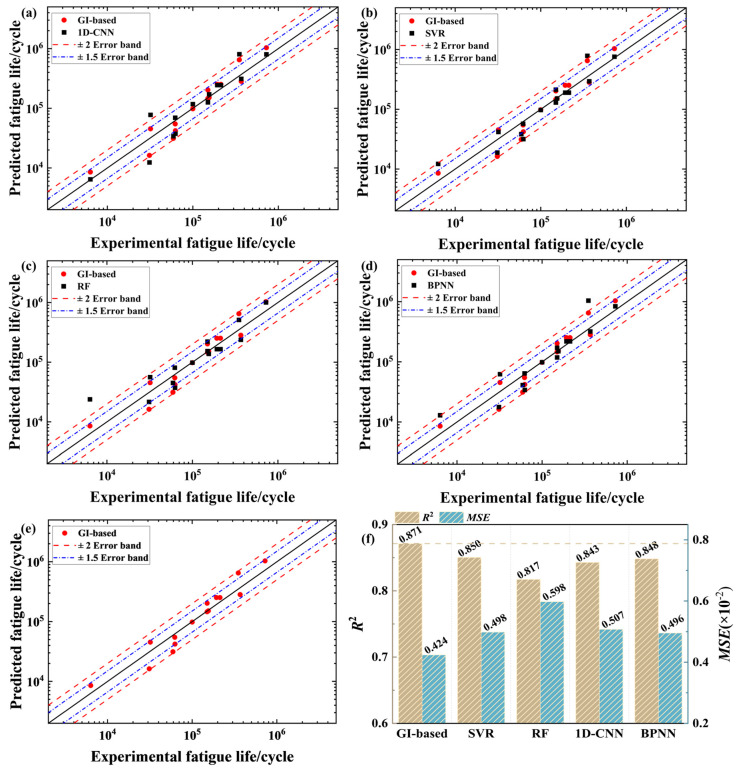
Comparison of experimental and predicted lives for LPBF AlSi10Mg alloy based on (**a**) 1D-CNN, (**b**) SVR, (**c**) RF, (**d**) BPNN, and (**e**) GI-based. (**f**) *R*^2^ and *MSE* values for each model.

Figure 11 further compares the experimental lives of the LPBF AlSi10Mg alloy with predictions from different models. In these figures, all evaluated models show a slight accuracy deterioration. This can also be observed in Figure 11f, where all evaluated models lead to lower *R*^2^ values and higher *MSE* values when compared with those recorded in Figure 10f. The phenomenon of accuracy deterioration can be attributed to the event that the LPBF AlSi10Mg alloy database involves more influential features, increasing the complexity of fatigue life prediction. Despite this, as shown in Figure 11, the GI-based model still yields the best accuracy among the evaluated models, with most predictions falling within the ×2 error band. Such a good prediction performance well evidences the superiority of the GI-based model against conventional ML models for life-prediction issues.

#### 3.3.2. Examination of the Contributors to the Prediction Performance

In this subsection, ablation studies are conducted to identify the factors governing the prediction performance of the GI-based model. To achieve this, two different models were designed by degrading the GI-based model to varying degrees. Their main components are listed in Table 3. The GI-A model exclusively uses tabular data with numeric features for training and predicting, and it does not include the SE attention mechanism. On this basis, the GI-B model takes GIs as inputs. The other settings of the two degradation models remain consistent with that of the GI-based model.

**Figure 12:**
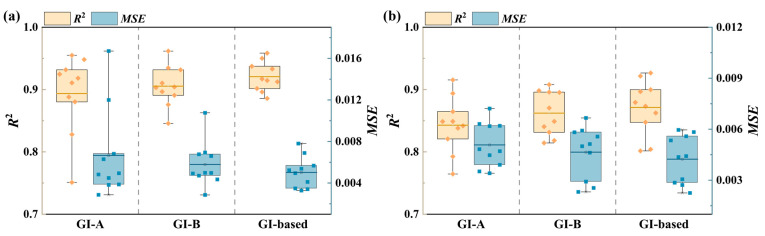
Comparison of prediction performance across models for (**a**) LPBF 316L stainless steel, and (**b**) LPBF AlMg10Si alloy.

Figure 12 displays the prediction performance of the GI-A, GI-B, and GI-based models across 10 repeated runs, with the statistical results summarized in Table 4. In Figure 12, each box bounds relevant data in the first and third quartiles, the hollow symbols inside each box signify the average of the related data, and the whiskers of each box represent the minimum and maximum of the related data. Moreover, in Table 4, m(⋅) and std(⋅) denote the average and standard deviation of bracketed variables, respectively, and their increments are represented by Δm(⋅) and Δstd(⋅), respectively.

It is evident in Figure 12a that for the LPBF 316L stainless steel, the average of the *R^2^* values gradually increases from the GI-A, GI-B, to GI-based models, while the average of *MSE* values changes conversely. Both phenomena underscore the benefits of GIs and the SE attention mechanism in improving prediction accuracy. In addition, it can also be seen that the variability of predictions from 10 runs is reduced progressively from the GI-A, GI-B, to GI-based models, showcasing an improvement trend in prediction stability. For the LPBF AlSi10Mg alloy, as shown in Figure 12b, the GI-based model still outperforms the GI-A and GI-B models in terms of prediction accuracy and stability. Nevertheless, it can be found in Figure 12b that there exist two instances where the *R*² value from the GI-based model is slightly lower than those from the GI-B model. These anomalies are raised possibly because the SE attention mechanism increases the model’s dependency on specific features, potentially compromising the prediction accuracy when facing certain data distributions [28]. The quantification results in Table 4 support the above observations.

One can conclude from the above results that the GI-based model stands out for using GIs as inputs, and it can be further enhanced by integrating the SE attention mechanism. The developed GI construction workflow produces highly informative GIs from the given tabular data, with information enhancement by the SHAP and PCC analyses, facilitating better data-driven modeling of fatigue life. On the other hand, the SE attention mechanism can help identify the most critical parts of GIs, ensuring the capture of essential data information. The combined use of GIs and the SE attention mechanism makes the GI-based model a promising tool for life-prediction tasks, though further examinations are necessary in the future.

#### 3.3.3. Comparative Analysis of Feature Maps with Attention Weights

To validate the benefits of the SE attention mechanism and gain more insights into the GI-based model, feature maps with attention weights are discussed in this subsection. Feature maps are visual representations of graphical features extracted by different CNN layers, which can be visualized as heatmaps. Areas with a higher color intensity in these heatmaps indicate stronger activations, highlighting the regions where the model focuses most intensely. They can reflect the model’s understanding of graphical features at various levels and also uncover the feature importance and intercorrelation [49]. Thus, they are certainly helpful in understanding how the GI-based model extracts, processes, and interprets information from input images across different CNN layers.

**Figure 13:**
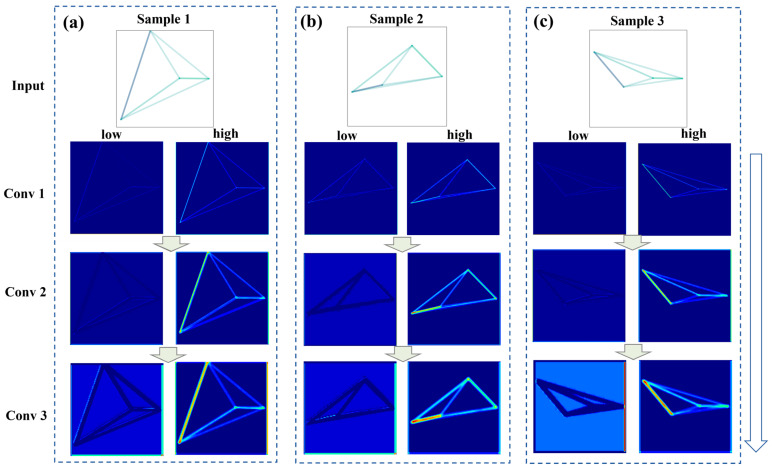
Comparative analysis of feature maps generated through different convolutional layers with high and low attention weights for three samples of LPBF 316L stainless steel: (**a**) sample 1, (**b**) sample 2, (**c**) sample 3. The input images (top row) are processed through three convolutional layers. Feature maps on the left correspond to low attention weights, while those on the right correspond to high attention weights.

**Figure 14:**
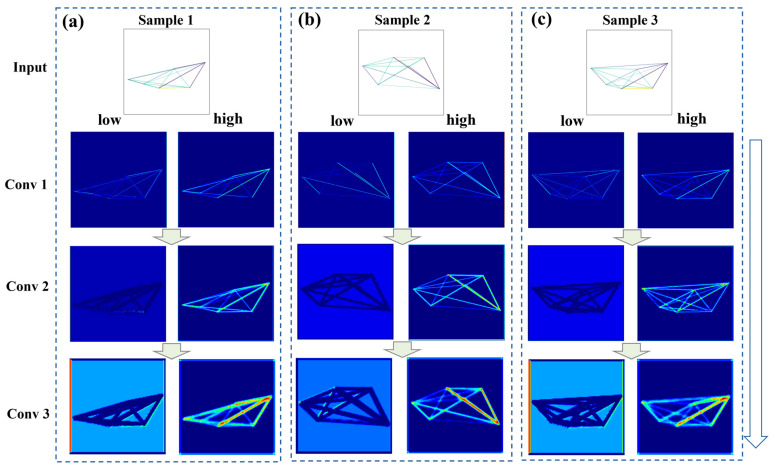
Comparative analysis of feature maps generated through different convolutional layers with high and low attention weights for three samples of LPBF AlMg10Si alloy: (**a**) sample 1, (**b**) sample 2, (**c**) sample 3.

To facilitate a more intuitive analysis, a comparative illustration is conducted between feature maps selected from the top 20% (high attention weights) and bottom 20% (low attention weights) across different convolutional layers. This helps select representative feature maps and provides a clear basis for evaluating the SE attention mechanism. As shown in Figure 13a–c, feature maps with higher attention weights exhibit clearer contours of graphical features compared to those with lower attention weights from the same layer and originating from the same GI. This distinction is particularly pronounced in Sample 2, as depicted in Figure 13b. Additionally, as shown in Figure 14a–c, the feature maps with high attention weights in Conv 3 display more pronounced differences between various parts of the feature maps. These clearer and more distinctive feature maps lead to a more precise data representation, confirming the positive impact of the SE attention mechanism on the GI-based model.

From Conv 1 to Conv 3, feature representations become increasingly distinct, with high attention weight maps showing clearer and more defined outlines, highlighting the attention mechanism’s role in enhancing key features.

Observing the feature maps with high attention weights from top to bottom in Figure 13 and Figure 14, it is evident that the graphical features’ shapes are distinctly captured. From Conv 1 to Conv 3, the feature maps reveal increasingly detailed and differentiated representations of the graphical features. In Figure 13, the feature maps of the LPBF 316L stainless steel show stronger responses on the left side, while in Figure 14, those for the LPBF AlMg10Si alloy are more concentrated on the right side. This suggests that the GI-based model can not only distinguish the shapes of graphical features, but also assign varying levels of importance to different parts of the inputted GIs. Additionally, by analyzing the commonalities of the high attention weight feature maps from Figure 13 and Figure 14, it is evident that the maps respond more strongly to the connecting lines in the graphical features, particularly those representing higher feature correlations. This highlights the importance of considering feature intercorrelation in fatigue life modeling. However, numerical data often fail to reflect these correlations in an intuitive way, whereas image data can showcase them more clearly. In conventional feature engineering processes for ML models, features with high PCC values are often eliminated to reduce dimensionality, but this approach may not always align with actual needs. Instead, it may be more effective to allow the ML model to automatically process this information by directly incorporating it, which is a significant advantage of the GI-based method.

## 4. Conclusions

This work investigates the use of graphical features for data-driven fatigue life assessment. A novel workflow is presented to transform widely used tabular data into corresponding geometric images (GIs). These GIs not only capture feature intensities, but also visually reflect the information on feature importance rankings and intercorrelations, leading to more informative graphical features. Building on this, a fatigue life model is proposed, where the GIs are regarded as inputs and the 2D-CNN is combined with the SE attention mechanism as the life predictor. The model is experimentally validated using two different databases of LPBF-fabricated metals. Based on the results obtained, the following conclusions can be drawn.

(1) With the aid of the SHAP and PCC analyses, both the importance of multiple features and their interrelationships can be embedded into the GIs, and the resulting graphical features contain supplementary information for ML models to correlate with fatigue life;

(2) Compared to conventional ML models using tabular data, the proposed GI-based model showcases a better prediction accuracy for the LPBF 316L stainless steel and LPBF AlMg10Si alloy. Its strong performance demonstrates the effectiveness of using GIs as inputs to ML models;

(3) The ablation study demonstrates that both the proposed GI construction workflow and the SE attention mechanism significantly enhance the prediction performance. The GI workflow provides informative input representations, while the SE mechanism improves the model’s focus on critical parts of data information;

(4) Further analysis of feature maps validates the effectiveness of the SE attention mechanism in enhancing the GI-based model. It also highlights the importance of considering feature intercorrelations when modeling fatigue life affected by multiple interactional factors.

Looking forward, to generalize the model to different materials or production processes, we plan to implement three key measures: (1) expanding the dataset to include additional materials, allowing for a broader scope of data to train the model and better understand how the GI-based approach applies across different materials, (2) defining core, universal features that capture fundamental fatigue characteristics common to various materials, which would help make the model adaptable to a wide range of materials without requiring extensive retraining, and (3) integrating transfer learning or active learning frameworks to leverage existing data and knowledge from related domains, enabling the model to better generalize to new, unseen materials or manufacturing processes. We plan to explore these strategies in future work to improve the model’s applicability to other materials and production methods.

Additionally, While the GI-based model offers a new perspective on fatigue life prediction by transforming tabular data into graphical representations, its computational cost remains a challenge due to the use of CNNs and the need for GPU resources. Future work will focus on exploring more efficient data transformation processes and lighter models to improve computational efficiency while maintaining predictive performance.

## Figures and Tables

**Table 1:** Processing conditions involved in established databases and corresponding sample sizes.

Materials	No.	*P*/W	*ʋ*/mm·s^−1^	*t*/μm	*h*/mm	Stress Ratio *R*	Number of Samples
LPBF AlSi10Mg alloy	1	250	500	50	0.15	0.1	26
	2	370	1300	30	0.16	0.1	4
	3	370	1300	30	0.19	0.1	4
	4	370	1300	30	0.22	0.1	4
	5	400	1000	30	0.2	−1	16
	6	100	930	50	0.1	−1	23
LPBF 316L stainless steel	1	98	1083	20	-	0.1	9
	2	126	853	20	-	0.1	7
	3	137	1083	20	-	0.1	14
	4	195	758	20	-	0.1	5
	5	195	1083	20	-	0.1	13
	6	195	1408	20	-	0.1	6
	7	254	1083	20	-	0.1	11
	8	264	1313	20	-	0.1	5
	9	293	1083	20	-	0.1	10
	10	195	1083	40	-	0.1	8
	11	195	1083	60	-	0.1	8
	12	195	1083	80	-	0.1	7

**Table 2:** Optimizing ranges of hyperparameters and resultant optimized values for five evaluated models.

ML Model	Hyperparameter	Optimizing Range	Optimized Value	
			LPBF 316L stainless steel	LPBF AlMg10Si alloy
BPNN	Number of hidden layers	[1, 2, 3, 4, 5]	3	5
	Number of neuron nodes in each hidden layer	[16, 32, 64, 128, 256]	32-64-32	64-128-128-128-64
SVR	C	[0.1, 1, 10, 100]	10 ‘rbf’	100 ‘poly’
	Kernel	[‘linear’, ‘poly’, ‘rbf’, ‘sigmoid’]		
	Gamma	[‘scale’, ‘auto’]	‘scale’	‘scale’
	Epsilon	[0.01, 0.1, 0.5, 1, 2]	0.01	0.01
1D-CNN	Kernel size	[2, 3, 4]	2	2
	Filters	[16, 32, 64, 128, 256]	64-128-64	32-64-32
	Layer	[1, 2, 3, 4, 5]		
RF	N_estimators	[10, 20, 40…640, 1280]	1280	320
	Min_samples_split	[1, 2, 5, 10, 20, 30]	2	2
	Max_depth	[2, 4, 6, 8, 10]	20	10
GI-based	Kernel size	[3 × 3, 5 × 5]	3 × 3, 3 × 3, 3 × 3	3 × 3, 5 × 5, 3 × 3
	Number of hidden layers	[1, 2, 3]	3	3
	Number of filters in each hidden layer	[16, 32, 64, 128, 256]	64-128-64	16-128-16

**Table 3:** Mode components devised for ablation study.

Model	GI	SE Attention Mechanism
GI-A	×	×
GI-B	√	×
GI-based	√	√

In the table, the symbols “√” and “×” represent the presence or absence of certain components in the models listed.

**Table 4:** Quantitative comparison of the prediction performance of different models.

Model/ Accuracy Index	LPBF 316L Stainless Steel				LPBF AlMg10Si Alloy			
	*m*(*R*^2^)	*m*(*MSE*)	*std*(*R*^2^)	*std*(*MSE*)	*m*(*R*^2^)	*m*(*MSE*)	*std*(*R*^2^)	*std*(*MSE*)
GI-A	0.8936	0.00666	0.0420	0.00125	0.8429	0.00507	0.0591	0.0041
Δm(⋅) or Δstd(⋅)	Baseline	Baseline	Baseline	Baseline	Baseline	Baseline	Baseline	Baseline
GI-B	0.9054	0.00579	0.0340	0.00145	0.8621	0.00465	0.0308	0.0020
Δm(⋅) or Δstd(⋅)	+0.0118	−0.00087	−0.008	+0.00020	+0.0192	−0.00042	−0.0283	−0.0021
GI-based	0.9206	0.00502	0.0414	0.00134	0.8713	0.00424	0.0224	0.0014
Δm(⋅) or Δstd(⋅)	+0.027	−0.00164	−0.0006	+0.00009	+0.0284	−0.00083	−0.0367	−0.0027

## Data Availability

The original contributions presented in this study are included in the article. Further inquiries can be directed to the corresponding author.
